# Insights into the potential dual-antibacterial mechanism of *Kelisha* capsule on *Escherichia coli*

**DOI:** 10.1186/s12906-024-04500-7

**Published:** 2024-05-28

**Authors:** Guolin Shi, Xiao Lu, Yuanhang Zheng, Tao Yang, Enyuan Zhu, Yanhong Song, Pintong Huang

**Affiliations:** 1 Department of Ultrasound in Medicine, School of Medicine, The Second Affiliated Hospital of Zhejiang University, Zhejiang University, 88 Jiefang Road, Shangcheng District, Hangzhou, 310009 China; 2 Post-Doctoral Research Center, Zhejiang SUKEAN Pharmaceutical Co., Ltd, Hangzhou, 311228 China; 3 Research Center of Ultrasound in Medicine and Biomedical Engineering, The Second Affiliated Hospital of Zhejiang University School of Medicine, Zhejiang University, Hangzhou, 310009 China; 4 Research Center for Life Science and Human Health, Binjiang Institute of Zhejiang University, Hangzhou, 310053 China

**Keywords:** *Kelisha* capsule, Natural medicine, Antibacterial mechanism, Network pharmacology, Molecular docking, ATP synthase

## Abstract

Traditional Chinese medicine (TCM), AYURVEDA and Indian medicine are essential in disease prevention and treatment. *Kelisha capsule* (*KLSC*), a TCM formula listed in the Chinese Pharmacopoeia, has been clinically proven to possess potent antibacterial properties. However, the precise antimicrobial mechanism of *KLSC* remained unknown. This study aimed to elucidate the dual antibacterial mechanism of *KLSC* using network pharmacology, molecular docking, and experimental validation. By analyzing the growth curve of *Escherichia coli* (*E. coli*), it was observed that *KLSC* significantly inhibited its growth, showcasing a remarkable antibacterial effect. Furthermore, SEM and TEM analysis revealed that *KLSC* damaged the cell wall and membrane of *E. coli*, resulting in cytoplasmic leakage, bacterial death, and the exertion of antibacterial effects. The network pharmacology analysis revealed that *KLSC* exhibited an effect on *E. coli* ATP synthase, thereby influencing the energy metabolism process. The molecular docking outcomes provided evidence that the active compounds of *KLSC* could effectively bind to the ATP synthase subunit. Subsequently, experimental findings substantiated that *KLSC* effectively suppressed the activity of ATP synthase in *E. coli* and consequently decreased the ATP content. This study highlighted the dual antibacterial mechanism of *KLSC*, emphasizing its effects on cell structure and energy metabolism, suggesting its potential as a natural antibacterial agent for *E. coli*-related infections. These findings offered new insights into exploring the antibacterial mechanisms of TCM by focusing on the energy metabolism process.

## Introduction

Bacterial infections have become a major factor affecting public health issues [1]. At present, the use of antibiotics is a simple and common treatment for infections. However, the increasing and widespread use of antibiotics exacerbated the development of drug-resistant bacteria. Drug-resistant bacterial infections cause approximately 700,000 deaths worldwide each year [2]. Drug-resistant pathogens have caused a serious threat to global health and antimicrobial treatment [3, 4].

To address an unfavorable situation, one way is to develop new and more potential antibacterial drug candidates. Recent studies have shown that newly synthesized cadmium (II) complexes exhibit strong antibacterial and antifungal effects [5]. Furthermore, iron nanoparticles synthesized using novel and green synthetic techniques have demonstrated potential antibacterial activity against *E. coli* in 2022 [6]. In 2023, a review elucidates in detail that some newly synthesized pyridine compounds show good antimicrobials and can inhibit multidrug-resistant bacteria [4]. Another approach is to find a promising alternative strategy for treating bacterial infectious diseases [7]. Combination drug therapy is one of the treatment methods. Encouragingly, the essential oil of *Copaifera spp.* presents a promising alternative treatment for infections caused by multidrug-resistant *Staphylococcus aureus* when combined with conventional antibiotics or used in conjunction with LED lights [8].

At the same time, traditional Chinese medicine (TCM) with multi-target antibacterial modes is emerging as one of the effective antibacterial agents against bacteria [9]. Numerous studies have highlighted the significant antimicrobial properties of natural compounds and medicinal plants [10–14]. In vitro experiments have conclusively shown that extracts of *Elatostema papillosum Wed.* exhibit bacteriostatic effects when using different extracting solvents [15]. Medicinal plants have been proven to damage the cell wall structure as part of their antibacterial mechanisms, as evidenced by numerous studies. *Rose* essential oil disrupted the cell walls and membranes of *Pseudomonas putida*, as observed through scanning electron microscope (SEM) and transmission electron microscope (TEM) observation [16]. This disruption caused the leakage of intracellular substances, thereby exerting antibacterial effects [17–19].

Affecting the energy metabolism process is another way for TCM to inhibit bacteria. Bacterial ATP synthase is a potential therapeutic target for developing a new solution to treat bacterial infections [20]. ATP synthase, also called F_0_F_1_ ATP synthase, plays a vital role in cellular bioenergetics by facilitating the production of ATP from ADP and inorganic phosphate (Pi) [21]. ATP energy is essential for the functioning of all organisms, and disruption of cellular bioenergetics could result in cell death [22]. For example, *Ocimum gratissimum L.* essential oil could exert an antibacterial effect by inhibiting ATPase activity and affecting its metabolic capacity [23]. And various natural active molecules have been proven to inhibit the ATP synthase activity of bacteria, such as resveratrol, thymoquinone and Quercetin [24]. However, there is limited research on how TCM inhibits bacterial ATP synthase activity or impacts the energy metabolism pathway in terms of its antibacterial mechanism.

*Kelisha capsule (KLSC)* is a Traditional Chinese Medicine (TCM) commonly used in China. Its potent antibacterial properties have been demonstrated, yet its specific antibacterial mechanism has not been thoroughly explored, particularly at the molecular level. Network pharmacology, a novel discipline for studying drug mechanisms [25], offers a valuable bioinformatics tool for elucidating the synergistic therapeutic effects of TCM [26, 27]. While existing literature predominantly focuses on drug mechanisms in the human body, this study employs network pharmacology in a novel manner to investigate the action mode of *KLSC* on bacteria, specifically *Escherichia coli* (*E. coli*).

To gain a comprehensive understanding of the antibacterial mechanism of *KLSC* against *E. coli*, the SEM and TEM were employed to observe the morphological alterations in *E. coli* cells. Subsequently, this study investigated the impact of *KLSC* on the *E. coli* energy metabolism process based on network pharmacology combined with molecular docking. The study revealed the dual antibacterial mechanism of *KLSC*, involving disruption of bacterial cell structure and energy metabolism pathways. The research findings contribute to exploring the antimicrobial mechanism of TCMs through the energy metabolism pathway, providing valuable insights into potential therapeutic strategies.

## Materials and methods

### Materials and reagents

The *KLSC* utilized in this study was provided by *Zhejiang Sukean* Pharmaceutical Co., Ltd. It consisted of a combination of 12 Chinese medicinal ingredients, which were finely powdered and encapsulated into capsules. The key ingredients of *KLSC* were the medicinal herb clove and Piper longum L, which were recognized as the official botanical remedies in AYURVEDA and the Indian medicine system. And the TCM information contained in *KLSC* was shown in Table 1. *Escherichia Coli* (BNCC360097) was purchased from BeNa Culture Collection. Luria-Bertani (LB) medium (Lot: 20,221,031) was obtained from Beijing Solarbio Science & Technology Co., Ltd.

**Table 1:** All ingredient information of *KLSC*

No.	Scientific species names	Proportion (%)
1	*Angelica dahurica* (Fisch.ex Hoffm.) Benth.et Hook.f.	18.43
2	*Acorus tatarinowii* Schott.	9.22
3	*Piper longum* L.	5.54
4	*Gleditsia sinensis* Lam.	9.22
5	*Eugenia caryophyllata* Thunb.	5.54
6	*Atractylodes lancea* (Thunb.) DC.	9.22
7	*Asarum heterotropoides* Fr. *Schmidt* var. *mandshuricum* (Maxim.) Kitag.	7.36
8	*Centipeda minima* (L.) A. Br. et Aschers.	5.54
9	*Realgar*	3.07
10	*Saltpeter*	7.36
11	*Alumen*	18.43
12	*Dryobalanops aromatica* Gaertn.f.	1.07

### Antibacterial effect of *KLS*C on *E. Coli*

#### Preparation of *KLSC* solution

The powdered *KLSC* was accurately weighed and transferred to a centrifuge tube. Subsequently, the LB medium was added, and the mixture was subjected to ultrasound treatment for 60 min. After the ultrasound, the solution was centrifuged at 10,000 rpm for 10 min. And the resulting *KLSC* solution was filtered through a 0.45 μm microporous membrane to obtain the final solution.

#### Determination of MIC and MBC

The minimum inhibitory concentration (MIC) was determined using the microdilution method [28, 29]. Two-fold serial dilutions of *KLSC* solution were prepared in LB medium in 96-well microtiter plates. Bacterial suspensions were added to each well. Next, the bacterial organisms were cultured for 18 h at 37 ℃ and 60% RH. After cultivation, 20 µL 5% 2,3,5-Triphenyl-2 H-Tetrazolium Chloride (TTC) solution was added to each well and cultured at 37 ℃ for 30 min in the dark. The viable *E.coli* cells could reduce the colorless TTC to pink or red 1,3,5-triphenyl formazan (TPF) [30]. Therefore, the MIC was identified as the lowest concentration at which no color change occurred.

To determine the minimum bactericidal concentration (MBC), the 100 µL *E. coli* organisms were spread onto LB medium plates and incubated for 24 h at 37 ℃ and 60% RH. The MBC was the lowest concentration at which no visible bacterial colonies were observed [31, 32].

### Measurement of the *E. Coli* growth curve

The bacterial growth curve was made to assess the impact of *KLSC* on *E. coli* growth [33, 34]. An overnight *E. coli* culture was added to the 48-well microtiter plate with varying concentrations of *KLSC* solutions. The *KLSC* solutions were at final concentrations of 1/2 MIC, MIC and 2 MIC. Next, the bacterial suspensions were inoculated at 37 °C and 60% RH. Finally, the growth of *E. coli* was monitored at different time points (0, 2, 4, 6, 8, 10, 12, and 24 h) by measuring the optical density at 600 nm (OD600) using a microplate reader (Tecan Spark). Control experiments were conducted using *E. coli* cells in the LB medium. All experiments were repeated three times.

### Effect of *KLSC* on the structure of *E. Coli*

#### Scanning electron microscopy observation

*E. coli* was grown in LB broth until reaching the logarithmic growth phase. Subsequently, the 20 mL *E. coli* suspensions were mixed with 20 mL *KLSC* solutions (128 mg/mL), and inoculated at 37 °C and 60% RH for 18 h. A control group was established by adding an equivalent volume of LB broth to *E. coli* suspensions. After cultivation, the *E. coli* cells from different experimental groups were collected by centrifugation at 4200 rpm for 4 min at 4 ℃ (Eppendorf centrifuge AG5424, Hamburg, Germany). The *E. coli* samples were prepared according to a published paper [35]. Then, the SEM (Hitachi SU8100, Japan) was applied to observe the morphology of *E. coli* samples.

#### Transmission electron microscopy (TEM) observation

For TEM observation, the *E. coli* cells were collected using the same method described in Sect. 2.4.1. The samples were processed following the procedure outlined in the literature [35], involving fixation, dehydration, embedding, slicing, and staining. Subsequently, the morphologies of various *E. coli* samples were analyzed by TEM (Hitachi HT7800, Japan).

#### Measuring permeability of cell wall and cell membrane

To estimate the permeability of the cell wall, alkaline phosphatase (ALP) leakage was utilized as an indicator to evaluate the bacterial cell wall damage [36]. The *E. coli* suspensions in the logarithmic growth phase were treated with *KLSC* solutions in MIC and 2 MIC concentrations, respectively. These bacterial suspensions were then incubated at 37 °C and 60% RH. At 0, 2, 4, 6, and 8 h, the *E. coli* suspensions were centrifuged, and the supernatants were collected. ALP assay kit (lot: 101822230103, Beyotime Biotech. Inc. Shanghai, China) was used to measure the ALP leakage in the supernatants at different time points. The LB medium was served as the normal control.

#### Detection of electrical conductivity

Furthermore, the detection of electrical conductivity was conducted to evaluate cytoplasmic leakage from the bacterial cells into the LB medium [37]. The experimental procedure involved obtaining *E. coli* suspensions in the exponential growth phase, treating them with *KLSC* solutions at 1 MIC and 2 MIC concentrations, and then incubating the suspensions at 37 °C and 60% RH. At specific time intervals, the bacterial suspensions were collected. Following centrifugation at 10,000 rpm for 10 min, the conductivity of the supernatant was measured by a conductivity meter (DDBJ-350, Hangzhou Qi Wei Instrument Co., Ltd. Hangzhou, China). The LB medium was considered as the normal control. All experiments were repeated three times.

### Effect of *KLSC* on the ATP metabolism process of *E. Coli*

#### Compounds screening and putative protein targets of *KLSC*

In the Traditional Chinese Medicine System Pharmacology Database (TCMSP, https://old.tcmsp-e.com/tcmsp.php#), the main chemical compounds of medicinal herbs were searched. The bioactive phytoconstituents of each herbal medicine were collected based on oral bioavailability (OB ≥ 30%) and drug-likeness (DL ≥ 0.18) [38, 39]. Active components of mineral medicine in *KLS*C were screened from the HERB database (http://herb.ac.cn/). And some active ingredients were supplemented by relevant literature. The protein targets that acted on *E. coli* were predicted according to the STITCH database (http://stitch.embl.de/). The information on protein targets such as gene names or gene IDs was transformed through the UniprotKB database (https://www.uniprot.org/). A network encompassing “drug-compound-target-*E. coli*” was constructed using Cytoscape 3.9.1 software, with key active compounds being identified based on their degree values [40].

#### Construction and analysis of protein-protein interaction network

The intersection protein targets between *KLSC* and *E. coli* were considered potential antibacterial targets. Next, the intersection targets were imported into the STRING database (https://cn.string-db.org/) to construct a protein-protein interaction (PPI) network. The resulting PPI network was saved in a TSV format file and further analyzed using Cytoscape 3.9.1. Finally, the hub gene within the network was identified by the MCODE plug-in in the Cytoscape software.

#### Gene ontology and KEGG pathway enrichment analysis

To delve deeper into the pathway and biological implications of the network, GO (Gene ontology) and KEGG (Kyoto Encyclopedia of Genes and Genomes) pathway analysis were conducted using the STRING database. GO enrichment analysis included Molecular function (MF), Biological Process (BP), and Cellular Component (CC) [41]. In the pathway enrichment analysis, the statistical significance threshold was set to *p* < 0.05.

#### Simulating validation of molecular docking

To confirm the interaction between active compounds of *KLSC* and *E. coli* protein targets, key proteins were used for molecular docking simulation. The active compound structures and core protein targets were obtained from PubChem (https://pubchem.ncbi.nlm.nih.gov/) and Protein Data Bank (PDB) (https://www.rcsb.org/), respectively. Following processing based on published papers [42, 43], Autodock Vina1.5.7 was employed for docking core targets with key active medicinal molecules. And the outcomes after molecular docking were visualized by PyMOL software.

### Measurement of ATP content

To validate the network pharmacology results, the intracellular ATP content of *E. coli* was measured. *E. coli* suspensions in the logarithmic growth phase were treated with *KLSC* solutions at MIC and 2 MIC concentrations, respectively, then incubated at 37 °C and 60% RH. Bacterial cells were collected by centrifugation at 4200 rpm for 10 min at 0, 0.5, 1.0, 1.5, and 2.0 h. Based on the instructions of the ATP Assay Kit (Lot: 072022220930, Beyotime Biotech. Inc. Shanghai, China), the intracellular ATP contents of *E. coli* were measured. The bacterial cells treated with LB medium served as the normal control group.

### Detection of ATP synthase activity

In order to explore the effects of *KLSC* on ATP synthesis, ATP synthase activity was detected. According to the culture method in Sect. 2.6, the *E. coli* cells treated with *KLSC* solutions were collected at various times. Then, the ATP synthase activities were detected according to the instructions of the ATP synthase Assay Kit (Lot: 20230625, Shanghai Jianglai Industrial Limited by Share Ltd, Shanghai, China). In this experimental test, the LB medium was considered as the normal control group.

### Data analysis

In this study work, all experiments were repeated three times. The experimental data were expressed as average values ± standard deviations. The significant differences were tested by One-way analysis of variance (ANOVA). And the threshold value for the significant level was set at *p* < 0.05.

## Results

### Antibacterial activity of *KLSC* against *E. Coli*

In this work, the MIC and MBC of *KLSC* against *E. coli* were initially determined using the microdilution method and TTC solution staining. The MIC was 64 mg/mL. After the bacterial suspensions were spread into LB medium plates for incubation, it was found that no bacterial colonies were grown at the concentration of 64 mg/mL. It indicated that the MBC was 64 mg/mL.

**Fig. 1:**
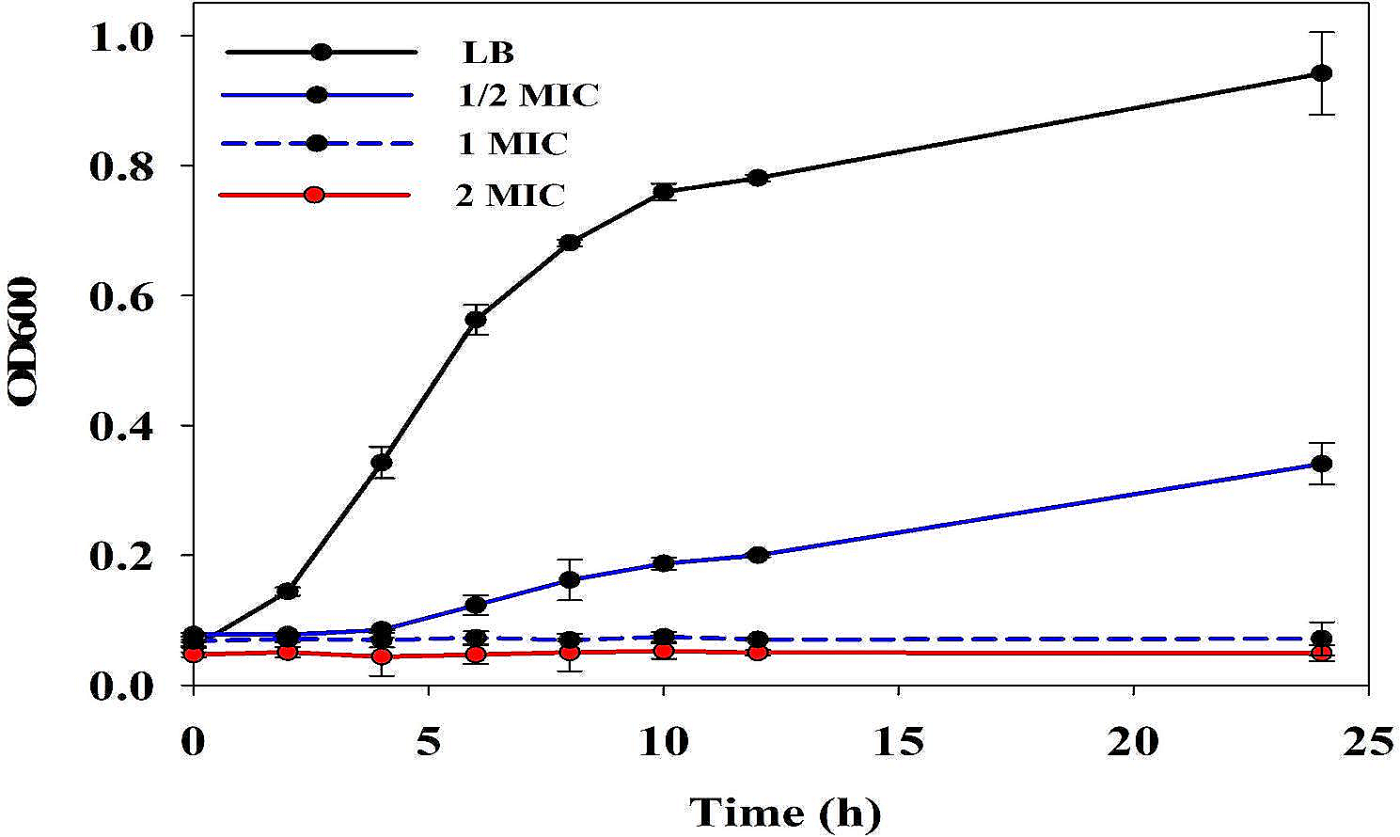
The growth curve of *KLS* capsule against *E. coli*

In order to further study the antibacterial activity against *E. coli*, the growth curves of *E. coli* were measured at 1/2 MIC, MIC and 2 MIC within 24 h. It could be found from Fig. 1 that the *E. coli* exhibited normal proliferation in the LB medium. However, the lag phase of the *E. coli* was postponed at 1/2 MIC, and the growth curve of *E. coli* decreased significantly. It was confirmed the inhibitory effect of *KLSC* against *E. coli*. At 1 MIC and 2 MIC, the *E. coli* growth was completely inhibited. It demonstrated that the *KLSC* had significant antibacterial effects.

### Effect of *KLSC* on the structure of *E. Coli*

#### The integrity of *E. Coli* cell wall

**Fig. 2:**
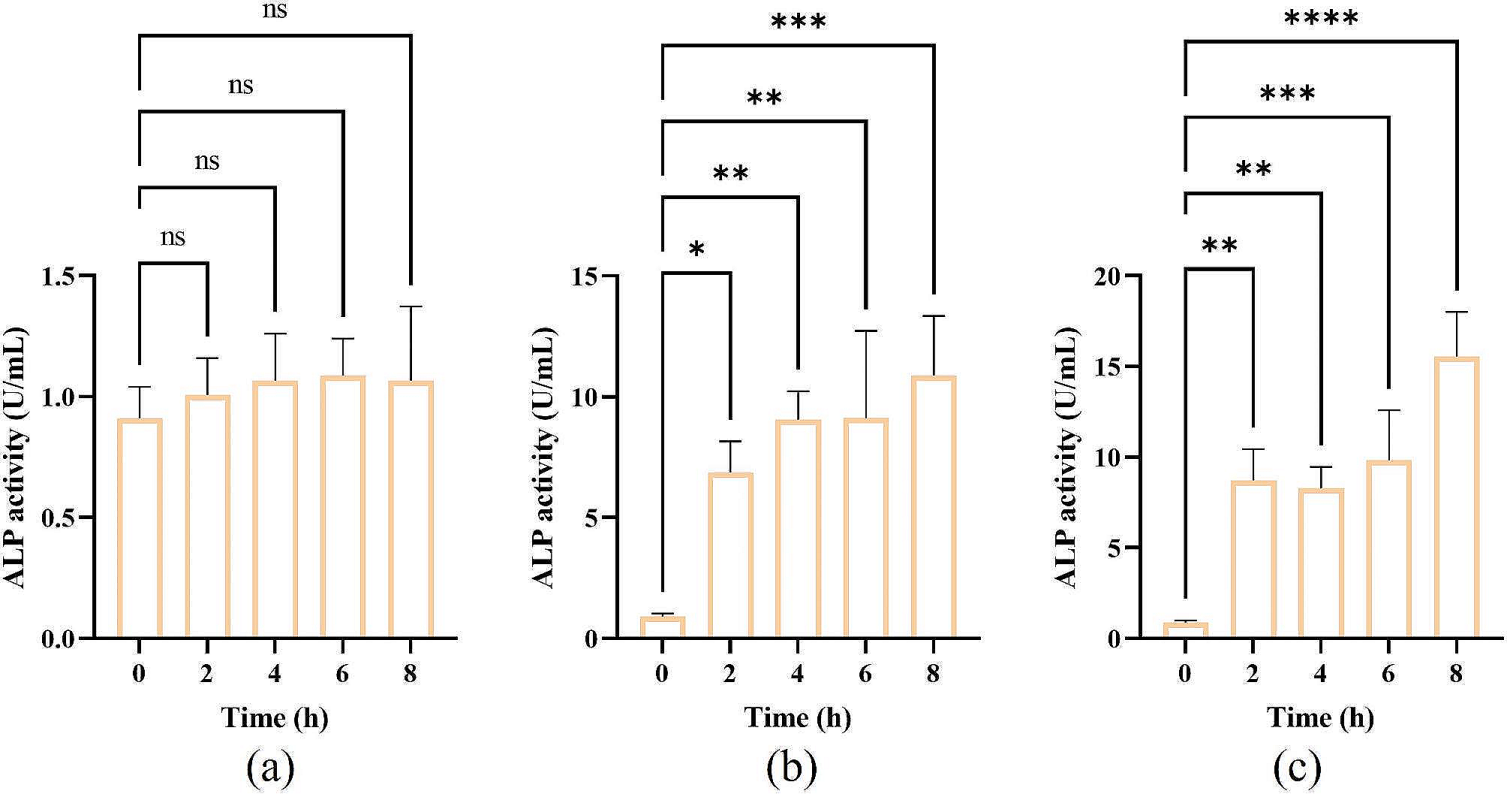
The ALP activity of *E. coli*. ((**a**) LB medium; (**b**) *KLSC* 64 mg/mL; (**b**) *KLSC* 128 mg/mL)

ALP, positioned between the cell wall and cell membrane, was not leaked into the extracellular environment [16]. Monitoring ALP activity in the extracellular environment served as an indicator of cell wall damage. The study measured ALP activity in the extracellular *E. coli*, with results depicted in Fig. 2. The ALP activity remained relatively stable in the LB medium for 8 h. It suggested that the *E. coli* cell wall maintained its normal structure. However, the ALP activity was significantly increased within 2 h at concentrations of 1 MIC and 2 MIC, respectively. It indicated that the *KLSC* could damage the cell wall within this timeframe. This damage led to the leakage of ALP into the LB medium, resulting in an elevation in extracellular ALP activity.

**Fig. 3:**
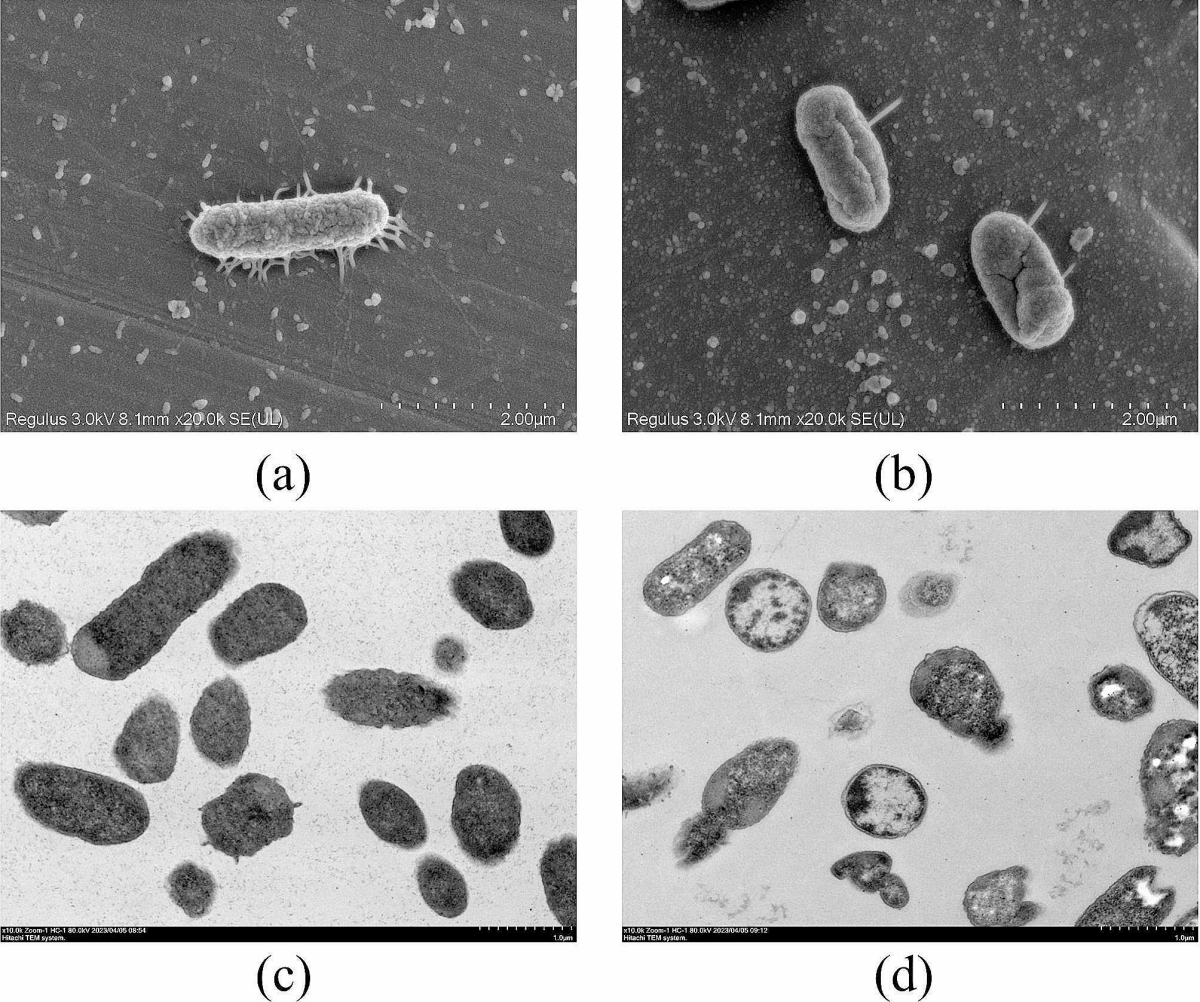
The structure of *E. coli*. (**a**) SEM micrographs of *E. coli* untreated with *KLSC*; (**b**) SEM micrographs of *E. coli* treated with *KLSC*; (**c**) TEM micrographs of *E. coli* untreated with *KLSC*; (**d**) TEM micrographs of *E. coli* treated with *KLSC*.

To visually assess cell wall changes, the SEM was used to examine the *E. coli* morphology. The results were displayed in Fig. 3. Figure 3 (a) showed that the untreated *E. coli* cells were intact and normal morphology, while those treated with *KLSC* showed evident differences. It could be seen from Fig. 3 (b) that the cell wall was cracked and damaged. The structure morphology was deformation and shrinkage. The morphological changes of *E. coli* were in accordance with the results of ALP activity. Based on the results of SEM and extracellular ALP activity, it was collectively confirmed that the *KLSC* could destroy the cell wall of bacteria, potentially leading to changes in the internal microenvironment and hastening bacterial cell death.

#### The integrity of *E. Coli* cell membrane

**Fig. 4:**
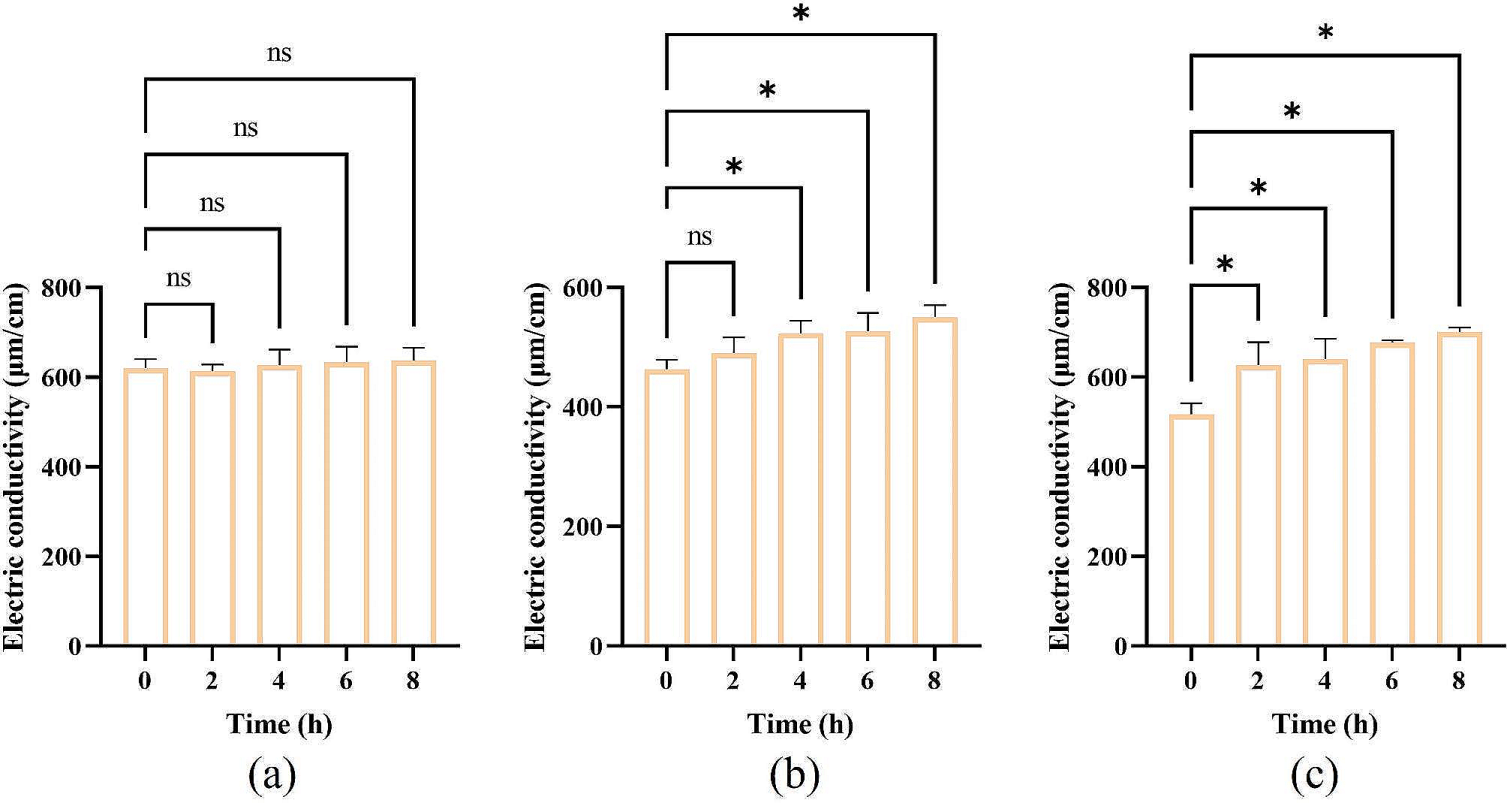
The extracellular relative electric conductivity. (**a**) LB medium; (**b**) *KLSC* 64 mg/mL; (**c**) *KLSC* 128 mg/mL

Maintaining the integrity of the cell membrane is one of the crucial conditions for the survival of bacterial cells [36]. The TEM images clearly illustrated that *E. coli* cells in the LB medium had intact cell structures (Fig. 3 (c)). The cytoplasm presented a dense and homogeneous state. Conversely, in the *KLSC* group (Fig. 3 (d)), intracellular cavities were observed, indicating cytoplasm leakage. Therefore, cytoplasmic leakage was bound to alter the conductivity of the LB medium, as Fig. 4 evidenced by a significant increase in conductivity in the *KLSC* group compared to the normal group. The elevated conductivity indirectly suggested the leakage of various cellular components into the medium, implying irreversible damage to the *E. coli* cell structure caused by *KLSC*.

The cell membrane of bacteria serves as a crucial protective barrier that is essential for maintaining the viability and integrity of the cell. Disrupting the integrity of the cell wall and membrane can cause changes in membrane permeability, leading to the leakage of nucleic acids and proteins from the cell [44]. These changes disrupt the balance of the internal environment and physiological processes to some degree, ultimately resulting in a loss of cell viability and hastening the bacteria’s death. In essence, the findings suggest that *KLSC* has the ability to break down the cell wall of *E. coli*, representing one of its antibacterial mechanisms.

### Effect of *KLSC* on the energy metabolism of *E. Coli*

ATP is crucial for various biological cellular processes and is essential for the survival of *E. coli* [24]. This study employs network pharmacology to reveal the effect of *KLSC* on ATP metabolism in *E. coli*.

#### Identification of active components and hub targets

**Fig. 5:**
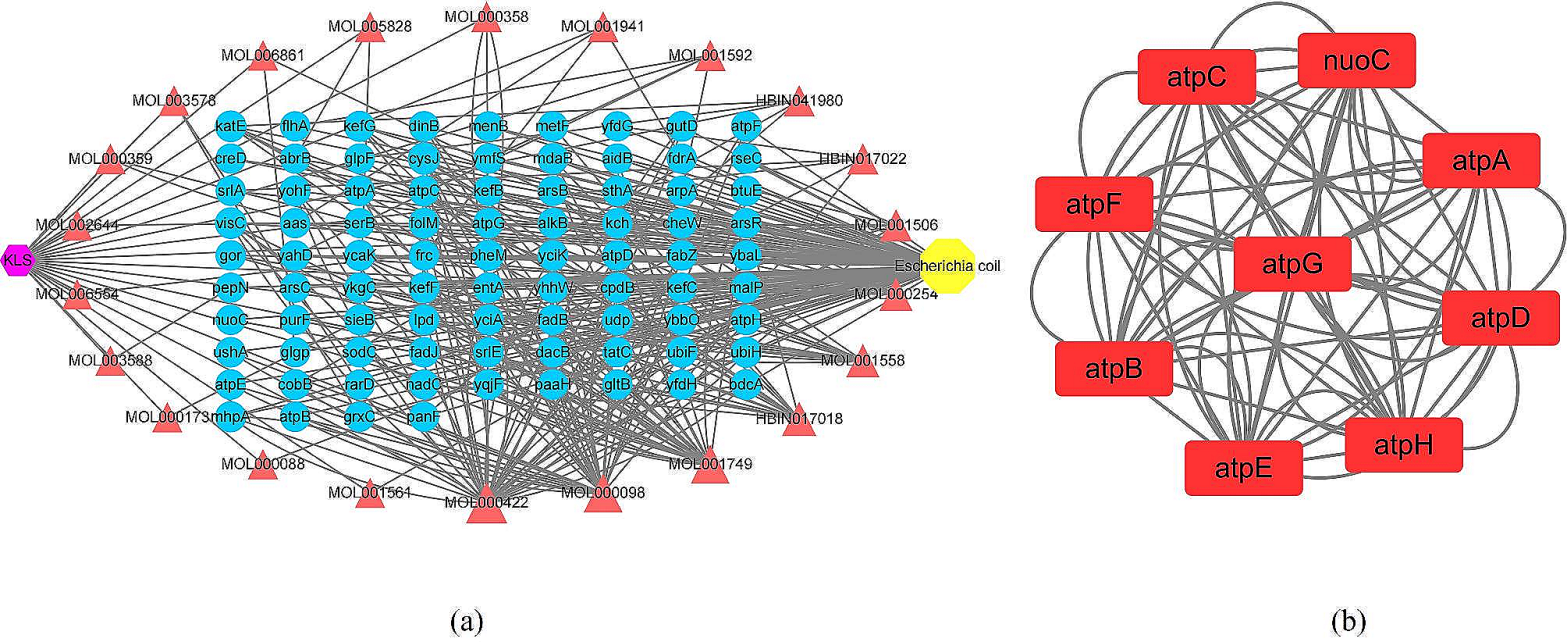
(**a**) The *KLSC* compound-target network. (**b**) The PPI networks

Initially, 82 active ingredients meeting the criteria of OB ≥ 30% and DL ≥ 0.18 were collected from the database. Since eugenol had an intense inhibitory activity against *E. coli*, it was added as an active pharmaceutical ingredient for network pharmacological analysis [45, 46]. Subsequently, a total of 83 active ingredients were examined, revealing that 22 of these interacted with 85 proteins in *E. coli*, as shown in Fig. 5 (a). Notably, the top ten compounds with larger degrees were identified as active components based on the “drug-compound-target-*E. coli*” network. The active molecules included kaempferol, bis(2-ethylhexyl) phthalate, quercetin, arsane, sesamin, eugenol, supraene, piperine, realgar, arsenic trisulfide, respectively. Detailed information on these active ingredients was listed in Table 2.

**Table 2:** The top ten active compounds of *KLSC*

TCM name	Degree	Molecular name
*Asari radix et rhizoma*, *caryophylli flos*, *acori tatarinowii rhizoma*	40	kaempferol
*Anglicae dahuricae radix*, *caryophylli flos*	31	bis(2-ethylhexyl) phthalate
*Caryophylli flos*, *Centipeda herba*	31	quercetin
*Realgar*	16	arsane
*Asari radix et rhizoma*, *caryophylli flos*	13	sesamin
*Caryophylli flos*	12	eugenol
*Anglicae dahuricae radix*	8	supraene
*Piperis longi fructus*	5	piperine
*Realgar*	5	realgar
*Realgar*	5	arsenic trisulfide

The hub genes associated with ATP metabolism, such as atpA, atpB, atpC, atpD, atpE, atpF, atpG, atpH, and nuoC, were identified through PPI network analysis using the STRING database and Cytoscape 3.9.1, as shown in Fig. 5 (b). These findings suggest that the active compounds in *KLSC* primarily interact with the ATP synthase of *E. coli*.

#### GO and KEGG analysis

**Fig. 6:**
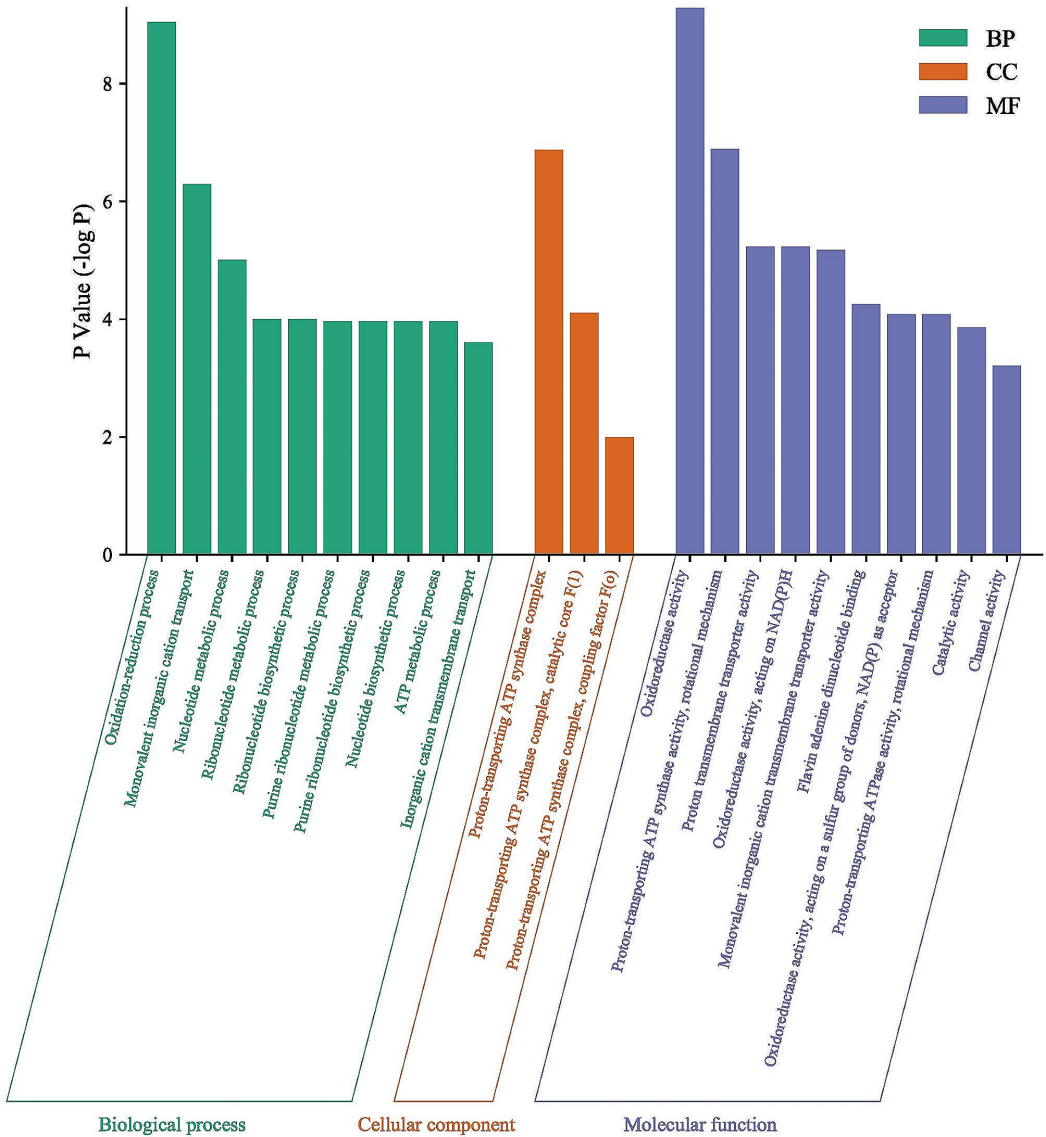
The GO enrichment analysis

In addition, the GO and KEGG enrichment analyses were conducted to investigate the underlying antibacterial mechanisms of *KLSC*. The GO enrichment was usually applied to analyze the functions of genes from the perspective of biological process (BP analysis), cellular component (CC analysis), and molecular function (MF analysis) [27]. Go analysis revealed that there were 23 items correlated with BP (*p* < 0.05) and three items CC (*p* < 0.05). The 24 items involved in MF (*p* < 0.05). And the results were displayed in Fig. 6. In the GO analysis, the CC was involved in the proton-transporting ATP synthase complex (GO: 0045259), proton-transporting ATP synthase complex, catalytic core F(1) (GO: 0045261) and proton-transporting ATP synthase complex, coupling factor F(0) (GO: 0045261). The BP was also involved in monovalent inorganic cation transport (GO: 0015672) and inorganic cation transmembrane transport (GO: 0098662). And the MF was involved in proton-transporting ATP synthase activity-rotational mechanism (GO: 0046933), proton transmembrane transporter activity (GO: 0015078) and proton-transporting ATPase activity-rotational mechanism (GO: 0046961). Based on the GO analysis, the *KLSC* could affect the activity of the ATP synthase complex through hindering the transmission of protons. Through KEGG enrichment analysis, the signaling pathways included seven items. As shown in Table 3, the pathways were related to metabolic pathways (eco01100), oxidative phosphorylation (eco00190), and tryptophan metabolism (eco00380). These results demonstrated that *KLSC* may exert antibacterial effects through various pathways and multiple targets.

**Table 3:** The KEGG enrichment analysis results

Term ID	Term description	False discovery rate
eco01100	Metabolic pathways	1.78 × 10^− 5^
eco00190	Oxidative phosphorylation	3.38 × 10^− 5^
eco00380	Tryptophan metabolism	0.0048
eco00130	Ubiquinone and other terpenoid-quinone biosynthesis	0.0329
eco00760	Nicotinate and nicotinamide metabolism	0.0416
eco00280	Valine, leucine and isoleucine degradation	0.0452
eco00362	Benzoate degradation	0.0477

#### Molecular docking investigation

**Fig. 7:**
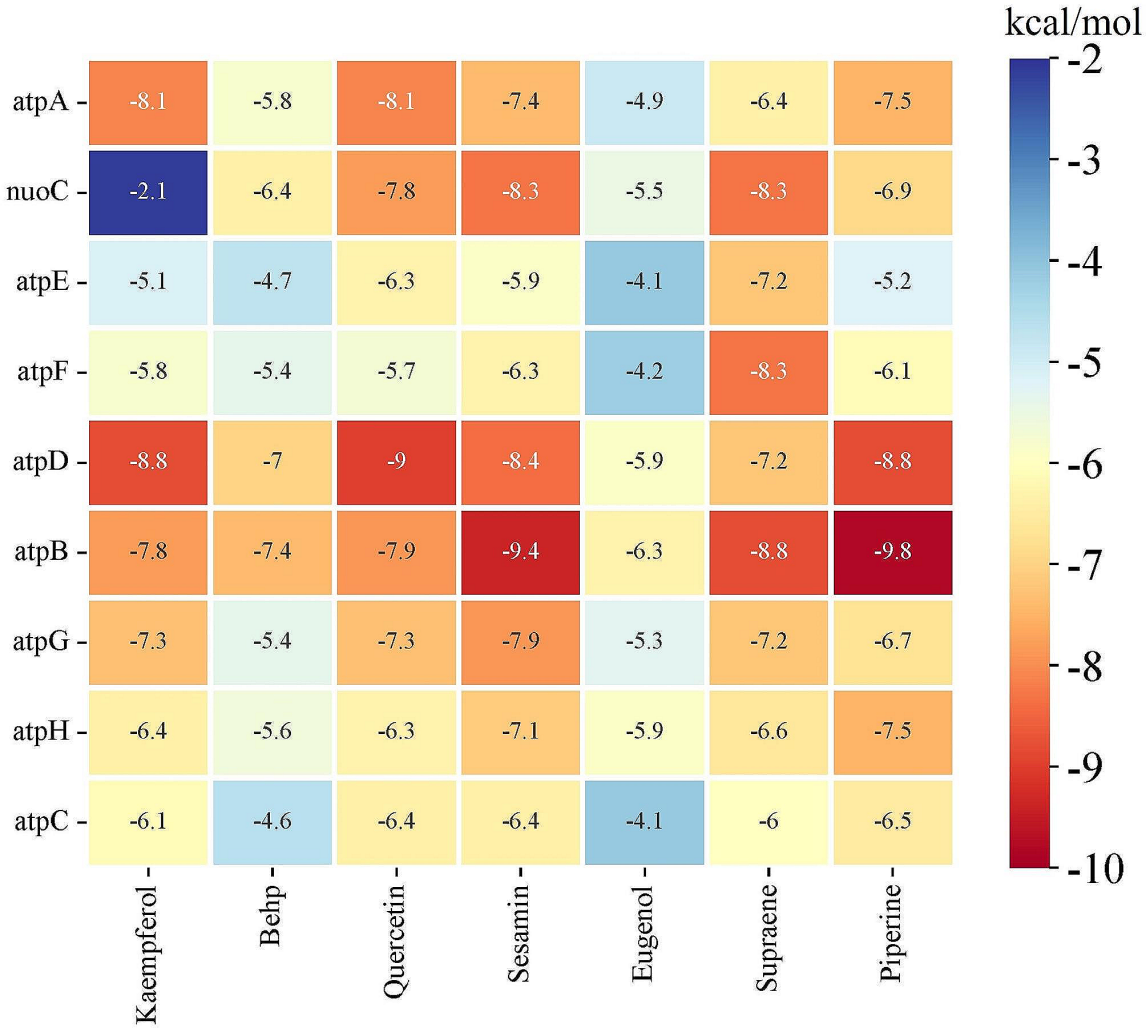
The heat map of Molecular docking binding energy. (Behp represents bis(2-ethylhexyl) phthalate)

Based on the findings from network pharmacology screening, the validation of interactions between active compounds and key targets was conducted using molecular docking. Generally, the binding energy between ligand and receptor was less than − 5 kcal/mol, indicating a good bind activity [25, 47]. Lower binding energies signified higher affinity and greater stability of the conformation. The results illustrated in Fig. 7 demonstrated that the binding energies between ligands and receptors were predominantly below − 5 kcal/mol. It indicated that these active compounds in *KLSC* had high affinities with the various ATP synthase subunits.

**Fig. 8:**
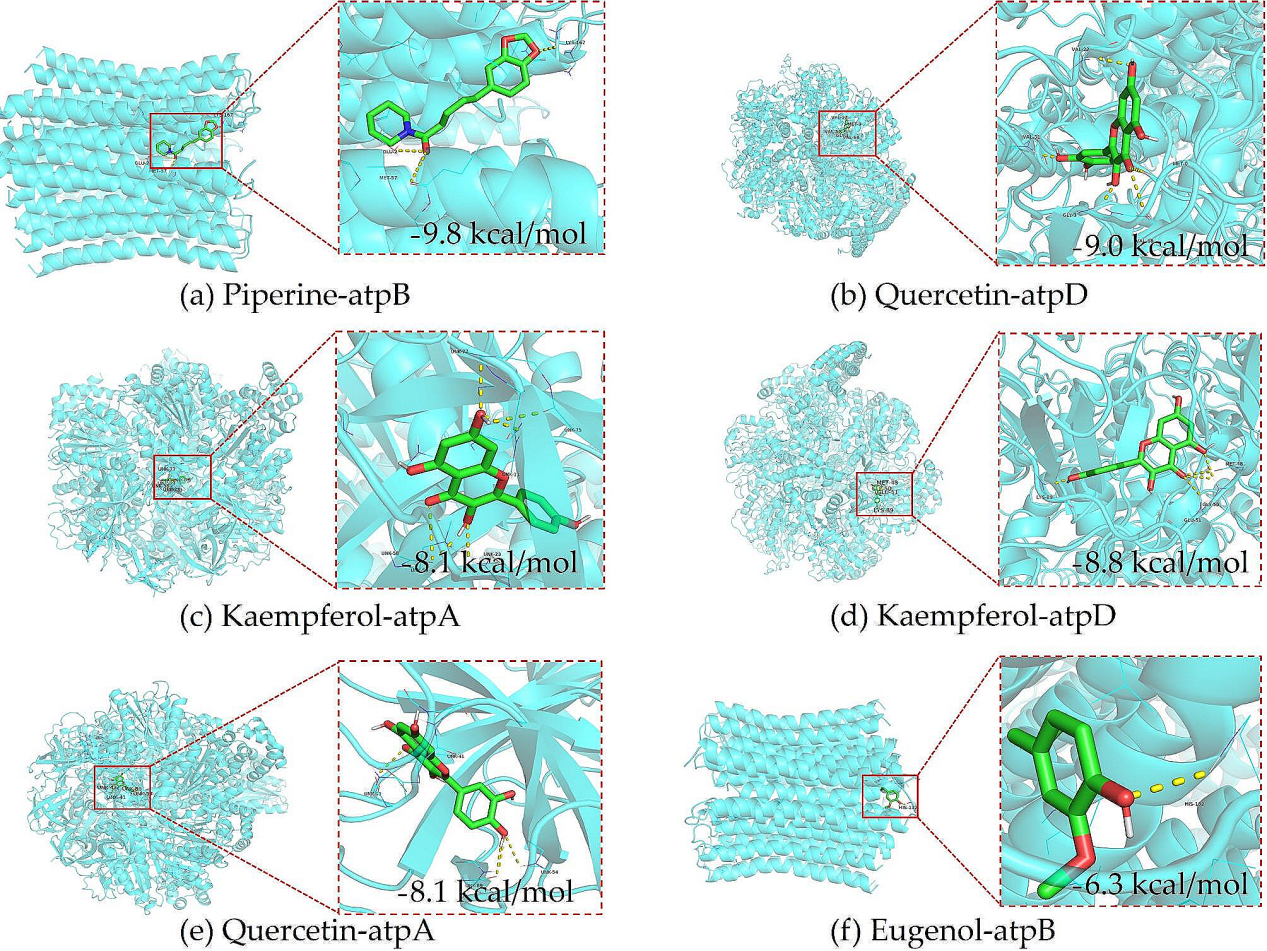
Molecular docking of active compounds and key targets

The molecular docking outcomes revealed that piperine exhibited the lowest binding energy (-9.8 kcal/mol) with atpB among all interactions, suggesting a strong interaction with atpB. Additionally, quercetin and atpD (-9.0 kcal/mol), kaempferol and atpA (-8.1 kcal/mol), kaempferol and atpD (-8.8 kcal/mol), quercetin and atpA (-8.1 kcal/mol), and eugenol and atpB (-6.3 kcal/mol) displayed notable binding affinities, as depicted in Fig. 8. These results indicated that the compounds in *KLSC* could effectively interact with ATP synthase subunits. Given that ATP synthase is crucial for ATP synthesis, the inhibition of ATP synthase activity by *KLSC* ultimately led to the death of *E. coli* cells. Furthermore, the integration of network pharmacology analysis suggested that *KLSC* might impact the energy metabolism process of bacteria by inhibiting the ATP synthase activity.

### Effects of ATP contents and ATP synthase activity

**Fig. 9:**
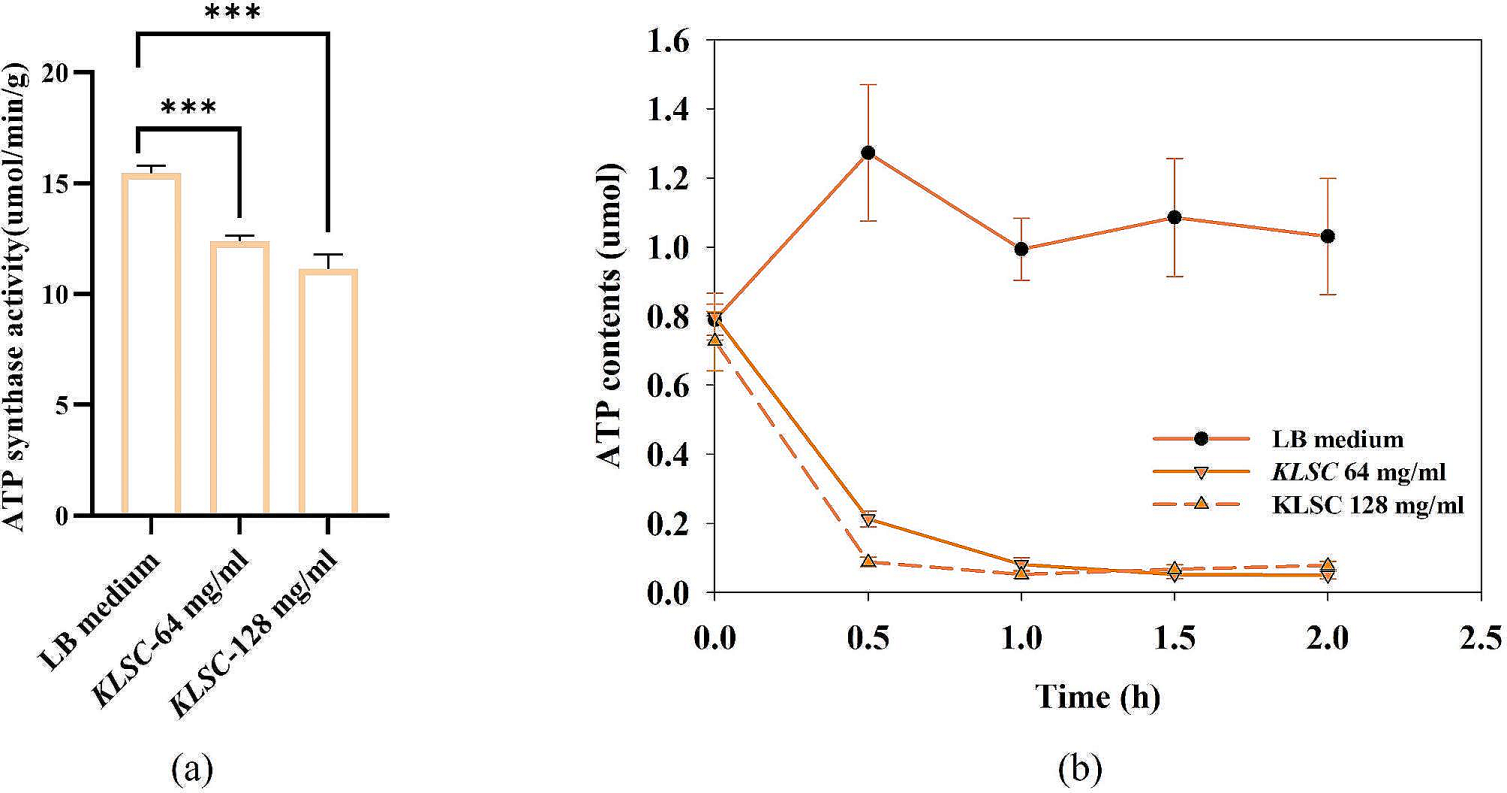
(**a**) The ATP synthase activity of *E. coli*; (**b**) The ATP contents of *E. coli*

Based on the outcomes derived from network pharmacology and molecular docking analyses, it was observed that *KLSC* had an impact on the ATP metabolism process. Subsequently, *KLSC* was utilized as the subject of experimentation to validate the predictions made through network pharmacology and molecular docking. As illustrated in Fig. 8, the bioactive components present in *KLSC* were found to interact with ATP synthase subunits in *E. coli*. In this section, the ATP synthase activity was measured based on the Assay Kit. It could be seen from Fig. 9 (a) that ATP synthase activity in *E. coli* cultured in the LB medium was 15.45 nmol/min/g. Conversely, the ATP synthase activities in *E. coli* cultured in MIC and 2 MIC *KLSC* were measured at 12.40 nmol/min/g and 11.15 nmol/min/g, respectively, with inhibitory rates of 19.74% and 27.83%. The decline in ATP synthase activity impeded ATP synthesis, thereby exacerbating bacterial mortality. Subsequently, the ATP levels within *E. coli* cells were assessed, with the results presented in Fig. 9(b). While a slight increase in ATP content was observed in the LB medium, likely due to active bacterial metabolism during the logarithmic phase, a substantial reduction in *E. coli* ATP content was noted in the presence of *KLSC* solution. This decline in ATP level was attributed to *KLSC*’s inhibition of ATP synthase activity, leading to reduced ATP production, as well as the disruption of the cell membrane system by *KLSC*, causing ATP leakage from *E. coli* cells. These findings underscore the significant reduction in ATP content induced by *KLSC*.

In conclusion, the collective evidence from network pharmacology, molecular docking, and experimental investigations indicated that *KLSC* could influence ATP synthase activity and ATP content. Given that ATP serves as the fundamental energy source in *E. coli*, ATP deficiency could result in cell death. Therefore, *KLSC* might exhibit antibacterial properties by modulating ATP synthesis.

## Discussion

TCM often contains multiple antibacterial components with multiple targets, and can exert its antibacterial effect through a combination of multiple processes. Therefore, bacteria are less likely to develop resistance to TCM. TCM plays a very important role in the treatment of drug-resistant bacterial infections [48]. As studied in this article, *KLSC* can damage the cell membrane system of *E. coli* and affect its energy metabolism process. Therefore, *KLSC* can exert antibacterial modes through the synergistic effect of multiple processes.

The maintenance of cell viability relies on the integrity of the cell wall. Disrupting the cell wall is a common antibacterial mechanism in traditional Chinese medicines (TCMs). For instance, compounds like eugenol and kaempferol have been discovered to possess antibacterial effects by destroying the cell wall [45, 49]. *KLSC* contains these active compounds as key components. Therefore, it can be inferred that some active molecules in *KLSC* have the potential to disrupt the bacterial cell wall. SEM and TEM studies support this hypothesis. The disruption of the bacterial cell wall by pharmacological molecules may involve the inhibition of penicillin-binding proteins (PBPs), which are crucial for peptidoglycan synthesis. Peptidoglycan is an essential component of bacterial cell walls [50]. The inactivation of PBPs can impede peptidoglycan formation, thereby hindering the development of cell walls [51] and ultimately leading to bacterial death. Therefore, PBPs are a major target for antibiotics [52]. Through molecular docking, a large number of papers have reported that natural active molecules can be bound to PBPs [53–56]. Natural active molecules like quercetin, eugenol and kaempferol have been reported to bind effectively to PBPs [57, 58]. It is worth noting that kaempferol exhibits higher specificity toward PBPs by interacting with crucial amino acids [59]. This suggests that the small molecules in *KLSC* have the potential to disrupt *E. coli* cell wall formation and exhibit antimicrobial properties by targeting PBPs. However, these hypotheses lack experimental validation, representing a limitation of this study.

**Fig. 10:**
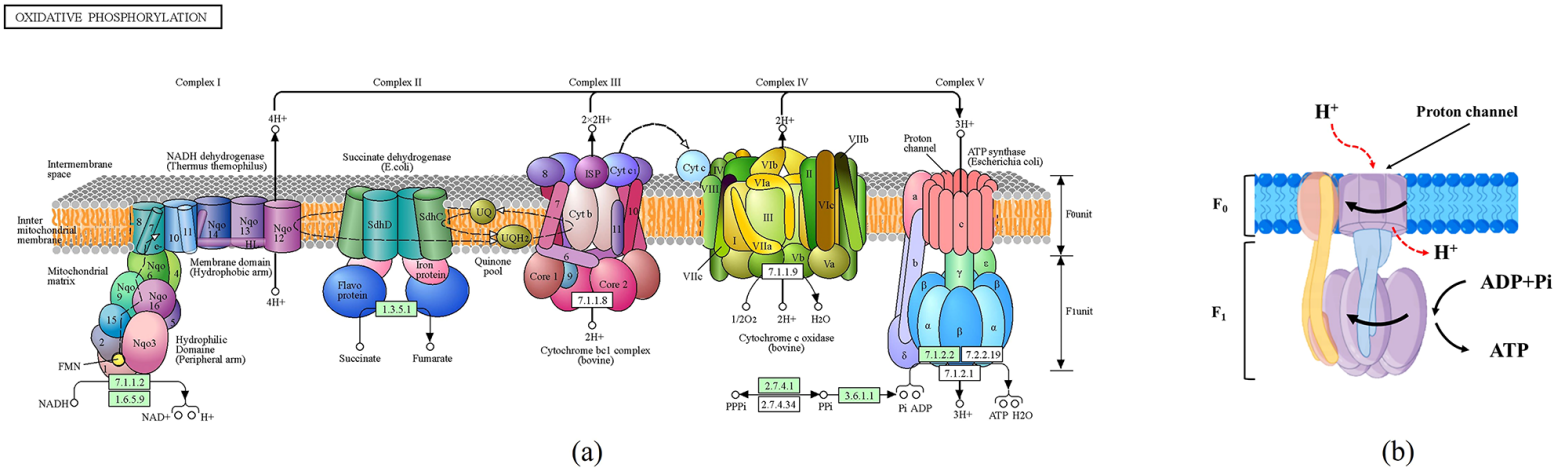
(**a**) The oxidative phosphorylation pathway of E. coli [62]; (**b**) ATP synthase of *E. coli*

ATP synthase, an extremely conserved enzyme embedded in the bacterial plasma membrane, is considered an effective pharmaceutical target for treating bacterial infections [60]. Energy metabolism plays a crucial role in an organism’s survival, molecular function, and biological process [61]. Oxidative phosphorylation is a key pathway for energy production, with ATP synthase being a critical component in this process, as shown in Fig. 10 (a) [62]. ATP synthase generates ATP during the terminal step of oxidative phosphorylation, which is crucial for various cellular functions [63]. Its inactivation can cause a reduction in ATP supply within bacterial cells, ultimately leading to bacterial death. Therefore, ATP synthase plays a vital role in bacterial survival. *KLSC* contains active compounds such as piperine, eugenol, and quercetin, which have been identified through network pharmacology analysis. Literature reports suggest that these compounds can inhibit ATP synthase activity to varying degrees [64–66]. Experimental evidence has shown that *KLSC* can inhibit ATP synthase activity and reduce intracellular ATP levels. Through network pharmacology, molecular docking, and literature findings, it is suggested that these active compounds may impact energy metabolism by targeting ATP synthase. For ATP synthase, proton gradient-driven clockwise rotation of the subunit is essential for ATP synthesis [67], as shown in Fig. 10 (b). Based on the GO analysis, *KLSC* may interfere with proton transmission, potentially disrupting proton gradients and impeding ATP synthase rotation, thereby reducing ATP synthesis. Antibacterial mechanism of *KLSC* involves the modulation of energy metabolism, as supported by the available evidence from network pharmacology and experimental studies.

Revealing the action mode of antimicrobial agents is an important prerequisite for understanding the mechanism of drug-resistant bacteria [5]. A promising solution for drug-resistant bacteria is the development of new antibacterial entities that act on novel targets of bacteria. Due to the different structures of ATP synthase between bacteria and mammalian [68], selectively targeting bacterial ATP synthase offers a safe and effective antibacterial approach that does not harm mammalian cells. Betaquiline is a successful ATP synthase inhibitor against multi-drug-resistant (MDR) and extensively drug-resistant (XDR) strains without causing toxic effects on ATP production in mammalian cells [20, 69]. Developing ATP synthase inhibitors is therefore viewed as a promising potential solution to overcome drug resistance. *KLSC* has multiple components and targets, and inhibits the activity of *E. coli* ATP synthase. Therefore, *KLSC* is a promising candidate for development as antibacterial agent against multidrug-resistant bacteria.

## Conclusions

In this work, it was shown to be remarkable antibacterial efficacy of *KLSC* against *E. coli*. *KLSC* treatment disrupted the cell wall and membrane structure of *E. coli*. This accelerated the leakage of intracellular substances, thereby inducing bacterial death. Additionally, network pharmacology and molecular docking proved that the *KLSC* exerted antibacterial effects through affecting the energy metabolism process. Experimental findings further confirmed that *KLSC* could inhibit ATP synthase activity and decrease intracellular ATP levels. Thus, the dual antibacterial mechanism of *KLSC* was elucidated, involving disruption of the membrane system and affecting the energy metabolism process.

## Data Availability

The data in the current study come from the TCMSP database (https://old.tcmsp-e.com/tcmsp.php), the STRING database (https://string-db.org/) and the HERB database (http://herb.ac.cn/). All data generated or analysed during this study are included in this published article.
